# Ophthalmic complications associated with COVID-19: a large US national database analysis

**DOI:** 10.1038/s41433-025-04050-3

**Published:** 2025-10-04

**Authors:** Alexander E. Azar, Priya Shukla, Kevin C. Allan, Rishi P. Singh, Katherine E. Talcott

**Affiliations:** 1 Case Western Reserve University School of Medicine, Cleveland, OH USA; 2 Cleveland Clinic Lerner College of Medicine of Case Western Reserve University, Cleveland, OH USA; 3 Center for Ophthalmic Bioinformatics, Cole Eye Institute, Cleveland, OH USA; 4 Cleveland Clinic Cole Eye Institute, Cleveland, OH USA; 5 Cleveland Clinic Martin Health, Stuart, FL USA

**Keywords:** Epidemiology, Risk factors, Eye diseases

## Abstract

**Objective:**

To assess ophthalmic complications following COVID-19 infection and the impact of mRNA vaccination.

**Methods:**

A retrospective cohort analysis was conducted using a large US database of de-identified electronic health records (1 March 2020–30 April 2021). Patients with COVID-19 and subsequent ophthalmology evaluation were included. The vaccinated COVID-19 cohort was matched to an unvaccinated cohort (n = 73,654 each). COVID-19 and influenza patients (n = 77,809 each) were also matched. Influenza served as a historical control. Ten ophthalmic conditions were assessed post-COVID-19 infection, including retinal artery occlusion (RAO), retinal vein occlusion (RVO), retinal oedema, vitreous haemorrhage, and neuro-ophthalmic manifestations. Bonferroni correction addressed multiple comparisons.

**Results:**

Vaccinated COVID-19 patients had significantly lower odds of retinal oedema (OR 0.68; 99.5% CI, 0.54–0.85), vitreous haemorrhage (OR 0.55; 99.5% CI, 0.44–0.68), and optic neuritis (OR 0.60; 99.5% CI, 0.43–0.85) compared to unvaccinated COVID-19 patients. There were no significant differences in the incidence of RAO, RVO, or retinal haemorrhage between vaccinated and unvaccinated cohorts. COVID-19 patients exhibited higher odds of diplopia (OR 1.89; 99.5% CI, 1.53–2.32) and cranial nerve VI palsy (OR 3.19; 99.5% CI, 1.82–5.59) compared to influenza patients, while rates of optic neuritis, RAO, RVO, retinal oedema, vitreous haemorrhage, and retinal haemorrhage were similar between the groups

**Conclusions:**

Vaccination was associated with a lower incidence of retinal oedema, vitreous haemorrhage, and optic neuritis. Compared to influenza, COVID-19 was associated with diplopia and CN VI palsy, while other neuro-ophthalmic and retinal pathologies had similar risk. Further research is needed to clarify these associations and underlying mechanisms.

## Introduction

Coronavirus disease 2019 (COVID-19) is a disease caused by the severe acute respiratory syndrome coronavirus 2 (SARS-CoV-2), which emerged in late 2019 in Wuhan, China, and led to a global pandemic [1, 2]. COVID-19 is characterised primarily as a respiratory illness, and patients commonly present with constitutional viral symptoms, such as cough, fever, fatigue, and sore throat [2]. In more severe cases, patients present with severe respiratory illness, including pneumonia and acute respiratory distress syndrome [1].

Though COVID-19 is primarily known for its respiratory effects, an evolving body of literature has demonstrated various ophthalmic manifestations of COVID-19. Common ocular symptoms that have been associated with COVID-19 include foreign body sensation, tearing, ocular irritation, redness, and chemosis [2–4]. Conjunctivitis is the most common ocular symptom related to COVID-19 and has been investigated as a potential early sign for COVID-19, especially due to the presence of the ACE2 receptor, the receptor where SARS-CoV-2 binds, on the conjunctiva and corneal epithelial cells [2, 5–8].

COVID-19 has also been associated with symptoms of the posterior segment of the eye, including retinal, vitreous, and optic nerve. Patients with COVID-19 infection have presented with central retinal vessel occlusions, vitreous haemorrhage, retinal haemorrhages, and retinal oedema [3, 9–11]. The mechanisms of posterior segment involvement in SARS-CoV-2 infection remain under investigation. Current evidence suggests that the virus can interact with the blood-retinal barrier, infect the retina, and induce retinal inflammation [12, 13]. Furthermore, various neuro-ophthalmic manifestations have been documented, including optic neuritis, ocular cranial neuropathy, cranial nerve palsies, nystagmus, and post-infectious demyelinating conditions [1, 3, 10]. Acute/subacute effects of COVID-19 include headache, insomnia, encephalitis, acute disseminated encephalomyelitis, and transverse myelitis [2, 3, 10]. COVID-19 has also been shown to lead to delayed effects to the central nervous system, including cognitive dysfunction, late ADEM, chronic headache, neuromyelitis optica spectrum disorder, and myelin oligodendrocyte glycoprotein antibody-associated disorders [9, 10, 14].

Though there are multiple case reports and meta-analyses investigating the ophthalmic complications of COVID-19, the sample sizes of the studies are generally small, and there is need for large-scale investigations to identify the incidence and risk of these manifestations within the population [2, 4, 15]. Capitalising on a large electronic claims platform, our study is uniquely positioned to identify ophthalmic manifestations related to COVID-19 infection. This population-level analysis enables more reliable identification of these associations, helping guide future research into their underlying mechanisms. Furthermore, our study provides a systematic comparison of vaccinated and unvaccinated COVID-19 patients, thus providing novel insights into the effects of mRNA vaccination on ophthalmic complications. Lastly, to address potential overrepresentation of rare outcomes in COVID-19-focused literature, we include a propensity-matched comparison of COVID-19 with influenza patients as a relevant, comparable viral illness.

## Materials and methods

A US collaborative network of aggregated, de-identified electronic health records of 107 million patients and 62 contributing healthcare organisations from 2006–2023 was examined for this study (TriNetX, Cambridge, MA). TriNetX, LLC is Health Insurance Portability and Accountability Act (HIPAA) compliant, certified to the ISO 27001:2013 standard, and maintains an Information and Security Management System to ensure protection of data. All data in the platform is de-identified and utilised to evaluate population level trends and thus for exemption from review by the Western Institutional Review Boards and qualified experts. Informed consent was neither possible nor necessary for this study. It was conducted in accordance with the Declaration of Helsinki.

Using a retrospective cohort study design, the platform was queried to generate four cohorts (Supplementary Table 1). A COVID-19 infected but vaccinated cohort was created by querying the database for mRNA vaccination *Common Procedural Technology* (CPT) codes and a documented COVID-19 infection between March 1, 2020 and April 30, 2021. This timeline was previously used in Ishayna et al. and Hebert et al. and encompasses the first 3 major COVID-19 pandemic waves [15, 16]. A COVID-19 infected but unvaccinated cohort was created by modifying identifying patients with a documented COVID-19 infection between March 1, 2020 and April 30, 2021 and no history of a COVID-19 mRNA vaccine. A third COVID-19-infected cohort was created with no limitations on vaccination status, but with the same timeframe limitation. A fourth historical control cohort was created by identifying patients who had a documented influenza infection before April 30, 2019 to ensure no patients had a COVID-19 infection. Influenza represents a clinically relevant comparator as it allows us to differentiate the ophthalmic sequelae specific to SARS-CoV-2 from those potentially attributable to general systemic viral illnesses. The date of April 30, 2019 precedes any known global circulation of COVID-19, which was first identified in late 2019. By restricting influenza diagnoses to this earlier timeframe, we ensured that none of the included patients had undocumented COVID-19 infection or were exposed to pandemic-related healthcare changes, thereby providing a true pre-pandemic control. This methodology has precedence in other large-scale EHR-based studies evaluating pandemic-related outcomes [15]. Furthermore, given that the influenza cohort was derived entirely from pre-pandemic data, these individuals had neither COVID-19 infection nor any exposure to COVID-19 vaccination. All cohorts had an inclusion criterion of the CPT code for Ophthalmology Services and Procedures (CPT 1012793) occurring after infection to ensure evaluation by an ophthalmologist. The look-back period began from the first recorded encounter in the TriNetX database. To ensure that the analysis only included new-onset ophthalmic outcomes, all cohorts were restricted to patients with no prior history of the respective ophthalmic diagnosis.

Vaccination status was determined based on mRNA vaccines (Pfizer-BioNTech and Moderna) due to their widespread use, distinct immunological mechanism, and the limited circulation of other vaccines in the United States, such as the Janssen (Johnson & Johnson) vaccine. Additionally, the Jannsen vaccine has been associated with thrombosis and thrombocytopenia, which could introduce confounding factors into our analysis [17–19]. This approach allowed us to minimise variability introduced by different vaccine platforms.

In order to account for potential hidden confounders, cohorts were matched using the propensity score analytic feature within the platform (greedy 1:1 matching, calliper of 0.25 SD). Cohorts were matched based on characteristics associated with higher risk for severe COVID-19 disease, according to the CDC’s list of ‘Underlying Conditions and the Higher Risk for Severe COVID-19’ [20]. Ten characteristics were chosen due to technical limitations within the platform, including age, sex, race, ethnicity, dyslipidaemia, diabetes, obesity, chronic obstructive pulmonary disease (COPD), chronic kidney disease (CKD), and asthma. International Statistical Classification of Diseases and Related Health Problems (ICD-10) codes for matching and analysis were used (Supplementary Table 2). Covariate balance among the propensity score matched cohorts was assessed with standardised mean differences (SMDs) where values greater than 0.10 were indicative of imbalance. Post-match SMDs were calculated, all of which were <0.1, indicating successful balance (Supplementary Table 3).

Eye outcomes were identified from the EHR using ICD-10 billing codes (Supplementary Table 2). Potential outcomes of interest were selected based on reports in the literature of retinal and neurological findings associated with COVID-19 [1, 3, 9, 10]. To qualify for the analysis, each participant must have been seen by an ophthalmologist and had a qualifying eye outcome, defined as having both a relevant ICD-10 code and an ophthalmology visit (CPT 1012793, Supplementary Table 2).

The incidence of new-onset ophthalmologic outcomes within 4 months of infection was then compared between cohorts. 4 months was chosen because odds of developing other acute manifestations of COVID-19, such as deep vein thrombosis and pulmonary embolism, are highest up to 110 days post-infection [21, 22]. Deep vein thrombosis (DVT) and pulmonary embolism (PE) were selected as positive controls for both analyses due to the well-established association between COVID-19 and venous thromboembolism [21, 23–25]. Previous studies have demonstrated an increased risk of venous thromboembolism in unvaccinated COVID-19 patients and all COVID-19 patients compared to influenza patients [22–27].

Resulting data showing the total patients with the outcome were reported and used to calculate cumulative incidence per 100,000. To account for multiple comparisons, statistical significance was determined using a Bonferroni correction to control the family-wise error rate. Results were considered statistically significant if the 99.5% confidence interval (adjusted for 10 comparisons) of the odds ratio excluded the null value of 1.00. All analyses were conducted using the built-in software of the TriNetX platform.

## Results

### COVID-19 vaccination vs. no vaccination

Vaccinated patients with COVID-19 infection and an ophthalmology visit (n = 74,976) were propensity score matched to 250,053 unvaccinated patients with COVID-19 infection and an ophthalmology visit. In the matched vaccinated group (n = 73,654) and unvaccinated group (n = 73,654), the mean age was 60.6 years, 61.6% were female, and 66.6% were White (Supplementary Table 3). In the matched unvaccinated group (n = 73,654), the mean age was 61.2 years, 62.8% were female, and 68.7% were White (Supplementary Table 3). The detailed results of the propensity score match, including the breakdown of race and comorbid factors, can be found in Supplementary Table 3.

Vaccinated patients demonstrated significantly lower odds of retinal oedema (OR 0.68, 99.5% CI 0.54–0.85), vitreous haemorrhage (OR 0.55, 99.5% CI 0.44–0.68), and optic neuritis (OR 0.60, 99.5% CI 0.43–0.85) compared to unvaccinated controls (Table 1). Differences in the incidence of retinal artery occlusion (RAO), retinal vein occlusion (RVO), and retinal haemorrhage were not statistically significant after applying the Bonferroni correction. The incidence of neuro–ophthalmic manifestations of COVID-19 was not significantly different between vaccinated and unvaccinated cohorts, apart from optic neuritis, which was more common in the unvaccinated group. Deep vein thrombosis (DVT) and pulmonary embolism (PE) were also more common in the unvaccinated cohort, which was expected and validates the study methods as these conditions served as positive controls [24, 25].

**Table 1:** Risk of ophthalmic manifestations in patients with SARS-CoV-2 mRNA vaccination compared to patients without vaccination.

Ophthalmic manifestation	Cohort	Patients in cohort	Patients with outcome	Incidence (per 100,000)	Odds ratio (Adjusted 99.5% CI)
RAO	COVID-19 vaccination	73,149	30	40	0.58 (0.31, 1.09)
	No vaccination	73, 127	50	70	
RVO	COVID-19 vaccination	72,618	50	70	0.64 (0.39, 1.05)
	No vaccination	72,662	80	110	
Retinal oedema	COVID-19 vaccination	71,833	120	160	**0.68 (0.49, 0.94)**
	No vaccination	72,012	180	240	
Retinal haemorrhage	COVID-19 vaccination	72,364	80	110	0.69 (0.46, 1.04)
	No vaccination	72,332	120	160	
Vitreous haemorrhage	COVID-19 vaccination	72,076	120	160	**0.55 (0.40, 0.75)**
	No vaccination	71,974	210	290	
Optic neuritis	COVID-19 vaccination	72,894	50	70	**0.60 (0.37, 0.98)**
	No vaccination	72,945	90	120	
Diplopia	COVID-19 vaccination	70,725	250	350	0.81 (0.64, 1.03)
	No vaccination	70,990	310	440	
CNIII palsy	COVID-19 vaccination	73,441	20	30	0.66 (0.29, 1.50)
	No vaccination	73,471	30	40	
CNIV palsy	COVID-19 vaccination	73,401	10	20	0.54 (0.21, 1.37)
	No vaccination	73,439	30	40	
CNVI palsy	COVID-19 vaccination	73,282	40	60	0.93 (0.51, 1.71)
	No vaccination	73,349	50	60	
DVT	COVID-19 vaccination	70,895	190	270	**0.73 (0.60, 0.88)**^**a**^
	No vaccination	70,937	260	370	
PE	COVID-19 vaccination	70,786	220	310	**0.75 (0.63, 0.89)**^**a**^
	No vaccination	71,140	300	420	

*SARS-CoV*-*2* Severe Acute Respiratory Syndrome Coronavirus 2, *mRNA* messenger RNA, *COVID-19* coronavirus disease 2019, *RAO* retinal artery occlusion, *RVO* retinal vein occlusion, *CN* *III* cranial nerve III, *CN IV* Cranial nerve IV, *CN VI* cranial nerve VI, *DVT* deep vein thrombosis, *PE* pulmonary embolism.

^a^DVT and PE were used as positive controls and not assessed for comparison, thus the standard 95% CI was utilised without need for Bonferroni correction.

The bold values indicate hazard ratios that remain statistically significant after Bonferroni correction for multiple comparisons, as evidenced by a 95% confidence interval that excludes the null value of 1.0.

In the vaccinated versus unvaccinated analysis, several outcomes showed nominally significant associations at the 95% confidence level that did not survive Bonferroni correction. These outcomes include RAO, RVO, retinal haemorrhage, and diplopia. For cranial nerve palsies (CN III/IV/VI), all 95% CIs included the null both before and after correction.

### COVID-19 vs. influenza

Patients with COVID-19 infection and an ophthalmology visit (n = 402,693) were propensity score-matched to 79,901 patients with influenza infection and an ophthalmology visit. In the matched COVID-19 group (n = 77,809), the mean age was 39.3 years, 58.8% were female, and 66.0% were White (Supplementary Table 4). In the matched influenza group (n = 77,809), the mean age was 39.7 years, 58.9% were female, and 65.1% were White (Supplementary Table 4). The detailed results of the propensity score match, including the breakdown of race and comorbid factors, can be found in Supplementary Table 4.

Compared to influenza patients, COVID-19 cases showed markedly higher odds of diplopia (OR 1.89, 99.5% CI 1.53–2.32) and cranial nerve VI palsy (OR 3.19, 99.5% CI 1.82–5.59; Table 2). However, there was no significant difference in the incidence of other neuro–ophthalmic manifestations, including optic neuritis, CN III palsy, or CN IV palsy, between the two cohorts. DVT and PE were included as positive controls, and both conditions were more common in the COVID-19 group, which was expected and validates the study methods [22, 23, 26, 27]. Differences in the incidence of retinal artery occlusion (RAO), retinal vein occlusion (RVO), retinal oedema, retinal haemorrhage, and vitreous haemorrhage were not statistically significant. Vitreous haemorrhage (OR 1.37, 95% CI 1.06–1.76) and CN III palsy (OR 2.33, 95% CI 1.19–4.59) demonstrated nominal significance at the 95% CI, but neither association retained statistical significance after Bonferroni correction.

**Table 2:** Risk of ophthalmic manifestations in patients with COVID-19 infection compared to patients with influenza infection.

Ophthalmic manifestation	Cohort	Patients in cohort	Patients with outcome	Incidence (per 100,000)	Odds ratio (99.5% CI)
RAO	COVID	77,538	20	30	0.84
	Influenza	77,540	30	30	(0.37, 1.93)
RVO	COVID	77,318	40	50	1.05
	Influenza	77,274	40	50	(0.55, 2.01)
Retinal oedema	COVID	76,840	100	140	0.85
	Influenza	76,967	120	160	(0.58, 1.24)
Retinal haemorrhage	COVID	76,943	90	120	1.29
	Influenza	77,035	70	90	(0.82, 2.03)
Vitreous haemorrhage	COVID	76,675	140	180	1.37
	Influenza	76,889	100	130	(0.95, 1.97)
Optic neuritis	COVID	77,168	80	100	1.04
	Influenza	77,202	70	90	(0.66, 1.65)
Diplopia	COVID	76,020	260	340	**1.89**
	Influenza	75,999	140	180	**(1.41, 2.54)**
CN III palsy	COVID	77,640	30	40	2.33
	Influenza	77,650	10	20	(0.88, 6.15)
CN IV palsy	COVID	77,582	30	40	1.65
	Influenza	77,604	20	30	(0.74, 3.66)
CN VI palsy	COVID	77,495	50	70	**3.19**
	Influenza	77,515	20	20	**(1.43, 7.13)**
DVT	COVID	76,058	140	190	**1.32**
	Influenza	75,800	110	140	**(1.03, 1.70)**^**a**^
PE	COVID	76,058	170	220	**1.64**
	Influenza	75,778	100	140	**(1.28, 2.09)**^**a**^

*COVID-19* coronavirus disease 2019, *RAO* retinal artery occlusion, *RVO* retinal vein occlusion, *CN* III cranial nerve III, *CN* IV cranial nerve IV, *CN* VI cranial nerve VI, *DVT* deep vein thrombosis, *PE* pulmonary embolism.

^a^DVT and PE were used as positive controls and not assessed for comparison, thus the standard 95% CI was utilised without need for Bonferroni correction.

The bold values indicate hazard ratios that remain statistically significant after Bonferroni correction for multiple comparisons, as evidenced by a 95% confidence interval that excludes the null value of 1.0.

## Discussion

This large-scale analysis advances our understanding of COVID-19’s ophthalmic manifestations by providing population-level risk estimates for both neuro-ophthalmic and posterior segment complications. Through comparison with influenza controls, we identify which manifestations reflect effects specific to SARS-CoV-2 versus general viral pathology. Furthermore, we offer evidence of mRNA vaccination’s potential role in modifying these ocular outcomes. The complications we assessed were rare, though our results showed an increased incidence of retinal oedema, vitreous haemorrhage, and optic neuritis in the non-vaccinated COVID-19 cohort. In the COVID-19 versus influenza comparison, diplopia and CN VI palsy were significantly more common in the COVID-19 cohort.

SARS-CoV-2 RNA has been found in the retinas of COVID-19 patients during autopsy, suggesting that the virus can have direct effects on the retina [28]. This is consistent with multiple reports of posterior segment haemorrhages, which are likely multifactorial and may be caused by inflammatory cytokines, a hypercoagulable state, and microvascular compromise induced by SARS-CoV-2 infection [11, 29–31]. The increased incidence of retinal oedema and vitreous haemorrhage in the non-vaccinated cohort suggests a potential for COVID-19 to affect posterior segment structures. This may be due to localised vascular or inflammatory changes driven by SARS-CoV-2 infection. The virus is known to cause endothelial injury, hyperinflammation, and microvascular dysfunction, all of which contribute to the hypercoagulability of COVID-19 and could predispose to vitreoretinal complications [11, 21, 29, 31–33].

Interestingly, despite the increased risk of thromboembolic events in COVID-19, which was validated by our positive controls (DVT and PE), there was no significant difference in the incidence of RVO or RAO between vaccinated and unvaccinated COVID-19 patients, as well as between COVID-19 and influenza patients. The lack of difference in RAO/RVO rates may stem from under-capture in EHRs due to the rarity of these events; however, there is a potential biologic basis. The latter hypothesis is supported by literature showing an uncertain relationship between systemic venous thromboembolism and coagulation disorders, despite the well-established association with atherosclerosis and cardiovascular disease [34, 35]. This is significant because it suggests that while systemic thrombogenic diseases like COVID-19 may increase the risk of large-vessel thromboembolism, the pathophysiology of RAO or RVO may involve distinct mechanisms.

SARS-CoV-2 has been also implicated in various neurologic manifestations, with one study finding that 36% of COVID-19 patients during the epidemic period in China had neurologic manifestations [36]. The increased incidence of neurologic findings in COVID-19 patients could potentially stem from two distinct mechanisms: hospitalisation effects or direct viral invasion. The hypercoagulable state associated with prolonged ICU stays and mechanical ventilation may lead to microvascular thrombosis or ischaemic injury affecting the cranial nerves. Secondarily, the ability of SARS-COV-2 to invade the central nervous system (CNS) has been speculated in the literature [37, 38]. The exact mechanism of CNS involvement is unclear, but may be related to the expression of ACE2 receptors in nerve cells, entry of the virus into the CNS through the cranial nerves, or systemic infection allowing it cross the blood brain barrier [39–41]. These processes may explain the greater risk of CN VI palsy and diplopia observed in the COVID-19 cohort compared to influenza. Many cases of CN palsy have been documented and a systematic review of 56 COVID-19 patients with cranial nerve palsy showed CN VII, VI, and III palsy were most common [38, 40–42]. Despite these mechanisms, our results showed no significant differences in the incidence of optic neuritis or other cranial nerve palsies between the COVID-19 and influenza cohorts.

Multiple rare cases of COVID-associated optic neuritis have been described in the literature, as well as reports of optic neuritis associated with COVID-19 vaccination [43–47]. The mechanism by which vaccination is not completely understood, but one possibility is that these patients have a pre-existing immunologic disease that is activated by immunisation [45, 48]. Our findings demonstrate that the incidence of optic neuritis is lower in vaccinated individuals. Influenza has been linked to neurologic abnormalities, including encephalopathy, cortical vision loss, and optic neuritis [49–51]. The similar incidence of optic neuritis in the COVID-19 and influenza cohorts may be due to shared immune-mediated or inflammatory processes triggered by viral infections. These findings highlight the complex interplay between systemic inflammation, vascular changes, and immune mechanisms in the development of neuro-ophthalmic complications in viral infections. While both viruses share some neurological effects, SARS-CoV-2’s distinct pathogenesis may explain these discrepancies in manifestations. More research is needed to clarify these specific CNS effects.

### Strengths and limitations

This study utilised a large sample size through a large electronic health record (EHR) platform, allowing for robust analysis of rare outcomes and the ability to generalise findings to the broader population. Furthermore, the use of propensity score matching helped to create comparable cohorts and mitigate the influence of confounding variables. We also included DVT and PE as positive controls to strengthen the internal validity of our study, as their association with COVID-19 is well-documented [21, 27, 52]. The analyses with influenza as a historical control and between vaccinated and unvaccinated groups also allowed for more robust comparison.

There were multiple limitations in this study. As an observational study, we cannot establish causal relationships between COVID-19 vaccination and the development of ophthalmic manifestations. Propensity score matching was done to help mitigate confounding factors, but this technique has many limitations. Propensity score matching alone does not address residual confounding, and the possibility of hidden confounders still exists and may have impacted our outcomes. Randomised control studies would better address these confounders, but they are impractical for rare outcomes such as those assessed here.

Many limitations pertain to the study’s reliance on an EHR platform. Since patients may obtain their care in multiple settings, each patient’s health care data may not be fully represented in the TriNetX platform. Vaccination status in particular may be underreported due to many individuals receiving COVID-19 vaccines in pharmacies or community centres that may not be directly documented in the EHR. Therefore, some vaccinated patients could have been improperly classified as unvaccinated, resulting in smaller differences between groups than what truly exists. In addition, individuals receiving pharmacy vaccinations may differ systematically from those vaccinated in EHR-linked facilities, introducing potential selection bias.

This study also relies on CPT and ICD-10 codes, which represents another limitation. Though they allow for quick and cost-effective analysis of millions of patients, they introduce the potential for misclassification bias and may overlook individual factors related to the patient’s clinical presentation.

Our results may have also been skewed by the ‘healthy vaccinee effect’ and ‘healthy user bias’ [53]. Individuals who opt for vaccination tend to be healthier due to proactive health behaviours or better access to healthcare. Therefore, the risk of ophthalmic manifestations in the vaccinated cohort could have been underestimated, as positive associations could have reflected overall better health rather than benefits of vaccination. While we did not perform additional subgroup analyses to account for healthy vaccinees, our propensity score matching adjusted for multiple comorbidities that may reflect overall health status.

This study reflects data from early COVID-19 strains (between March 1st, 2020 and April 30, 2021), and therefore may not generalise to newer COVID-19 or later vaccination phases. Additionally, multiple comparisons done within this study may have inflated false positive findings, though a Bonferroni correction was implemented to reduce type I error. While the Bonferroni correction reduces the likelihood of false positives, it may have masked clinically relevant associations that did not achieve statistical significance after adjustment.

Despite these limitations, this study provides valuable insights into the ophthalmic manifestations of COVID-19, highlighting both posterior segment and neuro-ophthalmic complications. Future studies using more comprehensive and granular datasets, including individual-level records, are necessary to confirm and expand upon these findings.

Supplementary information is available at *Eye’s* website.

## Summary

### What was known before

- COVID-19 has been linked to retinal vascular occlusions, optic neuritis, and neuro-ophthalmic complications.
- The impact of mRNA vaccination on reducing ophthalmic complications following COVID-19 infection is unclear.
- Limited large-scale studies have compared ophthalmic outcomes between COVID-19 and influenza infections.

### What this study adds

- In this cohort study of over 150,000 patients, COVID-19 vaccination was associated with lower odds of retinal oedema (OR = 0.68), vitreous haemorrhage (OR = 0.55), and optic neuritis (OR = 0.60).
- COVID-19 patients had higher odds of diplopia (OR = 1.89) and cranial nerve VI palsy (OR = 3.19) compared to influenza.
- The risk of retinal pathology was similar in COVID-19 and influenza patients.

## Supplementary information

Supplementary Table 1

Supplementary Table 2

Supplementary Table 3

Supplementary Table 4

## Data Availability

The data underlying this article were provided by TriNetX, LLC under license. Data cannot be shared publicly due to privacy laws and the proprietary nature of the TriNetX database. Researchers may request access to these data through a data use agreement with TriNetX at https://www.trinetx.com/.
