# Supplementary material for: Ophthalmic complications associated with COVID-19: a large US national database analysis
Source: Eye (Lond). 2025 Oct 4;39(17):3148–54. doi: 10.1038/s41433-025-04050-3 (PMC12623750; doi:10.1038/s41433-025-04050-3)
Supplement: Supplementary file 1 — Supplementary Table 1 [file 41433_2025_4050_MOESM1_ESM.docx]

**Supplementary Table 1.** TNX codes, CPT codes, and RxNorm codes were used to create cohorts of patients based on infection and vaccination status.

|  | Inclusion | Exclusion |
| --- | --- | --- |
| COVID-19 infected but vaccinated (1 March 2020 – 30 April 2021) | TNX:9088^1^  UMLS:CPT:91300  UMLS:CPT:91301  UMLS:CPT:0011A  NLM:RXNORM:2468231  UMLS:CPT:1012793^2^ |  |
| COVID-19 infected but unvaccinated (1 March 2020 – 30 April 2021) | TNX:9088  UMLS:CPT:1012793 | UMLS:CPT:91300  UMLS:CPT:91301  UMLS:CPT:0011A  NLM:RXNORM:2468231 |
| COVID-19 infected (1 March 2020 – 30 April 2021) | TNX:9088  UMLS:CPT:1012793 |  |
| Influenza infection (1 January 2000 – 30 April 2019) | UMLS:ICD10CM:J09  UMLS:ICD10CM:J10  UMLS:CPT:1012793 |  |

^1^TNX:9088 is a code specific to the TriNetX database and represents the presence of SARS-CoV-2 and related RNA. This was used to define COVID-19 infection.

^2^All cohorts had an inclusion criterion of the CPT code for Ophthalmology Services and Procedures (CPT 1012793) occurring after infection to ensure evaluation by ophthalmologist.

Abbreviations: TNX, TriNetX; CPT, Current Procedural Terminology; COVID-19, Coronavirus Disease 2019
