# Supplementary material for: Ophthalmic complications associated with COVID-19: a large US national database analysis
Source: Eye (Lond). 2025 Oct 4;39(17):3148–54. doi: 10.1038/s41433-025-04050-3 (PMC12623750; doi:10.1038/s41433-025-04050-3)
Supplement: Supplementary file 2 — Supplementary Table 2 [file 41433_2025_4050_MOESM2_ESM.docx]

**Supplementary Table #2.** ICD-10 codes used for analysis

| Disease | ICD-10 name | ICD-10 code |
| --- | --- | --- |
| Retinal artery occlusion (RAO) | Central retinal artery occlusion (CRAO) | H34.1 |
|  | Other retinal artery occlusions | H34.2 |
| Retinal vein occlusion (RVO) | Central retinal vein occlusion | H34.81 |
|  | Tributary (branch) retinal vein occlusion | H34.83 |
| Retinal oedema | Retinal oedema | H35.81 |
| Retinal haemorrhage | Retinal haemorrhage | H35.6 |
| Vitreous haemorrhage | Vitreous haemorrhage | H43.1 |
| Optic neuritis | Optic neuritis | H46 |
| Diplopia | Diplopia | H53.2 |
| CN III palsy | Third (oculomotor) nerve palsy | H49.0 |
| CN IV palsy | Fourth (trochlear) nerve palsy | H49.1 |
| CN VI palsy | Sixth (abducens) nerve palsy | H49.2 |
| Deep vein thrombosis (DVT) | Acute embolism and thrombosis of unspecified deep veins of lower extremity | I82.40 |
| Pulmonary embolism (PE) | Pulmonary embolism | I26 |
| Dyslipidaemia | Disorders of lipoprotein metabolism and other lipidaemias | E78 |
| Diabetes mellitus | Diabetes mellitus | E08-E13 |
| Overweight and obesity | Overweight and obesity | E66 |
| Emphysema | Emphysema | J43 |
| Other chronic obstructive pulmonary disease (COPD) | Other chronic obstructive pulmonary disease | J44 |
| Chronic kidney disease (CKD) | Chronic kidney disease | N18 |
| Asthma | Asthma | J45 |
| Pfizer SARS-CoV-2 mRNA Vaccine | Severe acute respiratory syndrome coronavirus 2 (SARS-CoV-2) (coronavirus disease [COVID-19]) vaccine, mRNA-LNP, spike protein, preservative free, 30 mcg/0.3 mL dosage, diluent reconstituted, for intramuscular use | 91300 |
| Moderna mRNA SARS-CoV-2 mRNA Vaccine | Severe acute respiratory syndrome coronavirus 2 (SARS-CoV-2) (coronavirus disease [COVID-19]) vaccine, mRNA-LNP, spike protein, preservative free, 100 mcg/0.5 mL dosage, for intramuscular use | 91301 |
| Moderna mRNA SARS-CoV-2 mRNA Vaccine | Immunisation administration by intramuscular injection of severe acute respiratory syndrome coronavirus 2 (SARS-CoV-2) (coronavirus disease [COVID-19]) vaccine, mRNA-LNP, spike protein, preservative free, 100 mcg/0.5 mL dosage; first dose | 0011A |
| SARS-CoV-2 mRNA vaccine | SARS-CoV-2 (COVID-19) vaccine, mRNA spike protein | 2468231 |

Abbreviations: ICD, International Statistical Classification of Diseases and Related Health Problems; CN, Cranial Nerve; SARS-CoV-2, Severe Acute Respiratory Syndrome Coronavirus 2; mRNA, Messenger RNA
