# Supplementary material for: Ophthalmic complications associated with COVID-19: a large US national database analysis
Source: Eye (Lond). 2025 Oct 4;39(17):3148–54. doi: 10.1038/s41433-025-04050-3 (PMC12623750; doi:10.1038/s41433-025-04050-3)
Supplement: Supplementary file 3 — Supplementary Table 3 [file 41433_2025_4050_MOESM3_ESM.docx]

**Supplementary Table 3**. Propensity score matching between vaccinated and unvaccinated COVID-19 groups

| Characteristic | Mean ± SD | Patients | % of Cohort | P-Value | Std diff. |
| --- | --- | --- | --- | --- | --- |
| Age at Index | 60.6 +/- 16.6 | 73,654 | 100% | <0.001 | 0.035 |
|  | 61.2 +/- 16.7 | 73,654 | 100% |  |  |
| White |  | 49,034 | 66.60% | <0.001 | 0.045 |
|  |  | 50,592 | 68.70% |  |  |
| Unknown Race |  | 2,372 | 3.20% | 0.022 | 0.012 |
|  |  | 2,219 | 3.00% |  |  |
| Female |  | 45,347 | 61.60% | <0.001 | 0.025 |
|  |  | 46,236 | 62.80% |  |  |
| Unknown Ethnicity |  | 7,813 | 10.60% | <0.001 | 0.044 |
|  |  | 6,852 | 9.30% |  |  |
| Not Hispanic or Latino |  | 59,443 | 80.70% | <0.001 | 0.051 |
|  |  | 60,905 | 82.70% |  |  |
| Hispanic or Latino |  | 6,398 | 8.70% | <0.001 | 0.025 |
|  |  | 5,897 | 8.00% |  |  |
| Black or African American |  | 13,937 | 18.90% | <0.001 | 0.033 |
|  |  | 13,011 | 17.70% |  |  |
| Other Race |  | 4,353 | 5.90% | <0.001 | 0.041 |
|  |  | 3,662 | 5.00% |  |  |
| Asian |  | 3,514 | 4.80% | 0.004 | 0.015 |
|  |  | 3,753 | 5.10% |  |  |
| Dyslipidaemia |  | 45,245 | 61.40% | 0.692 | 0.002 |
|  |  | 45,319 | 61.50% |  |  |
| Diabetes mellitus |  | 25,952 | 35.20% | 0.03 | 0.011 |
|  |  | 25,554 | 34.70% |  |  |
| Overweight and obesity |  | 29,007 | 39.40% | 0.011 | 0.013 |
|  |  | 28,530 | 38.70% |  |  |
| Emphysema |  | 3,969 | 5.40% | 0.417 | 0.004 |
|  |  | 3,899 | 5.30% |  |  |
| Other chronic obstructive pulmonary disease (COPD) |  | 8,723 | 11.80% | 0.13 | 0.008 |
|  |  | 8,536 | 11.60% |  |  |
| Chronic kidney disease (CKD) |  | 13,241 | 18.00% | 0.459 | 0.004 |
|  |  | 13,132 | 17.80% |  |  |
| Asthma |  | 15,655 | 21.30% | 0.017 | 0.012 |
|  |  | 15,281 | 20.70% |  |  |

Abbreviations: COVID-19, Coronavirus Disease 2019; COPD, Chronic Obstructive Pulmonary Disease; CKD, Chronic Kidney Disease
