# Supplementary material for: Ophthalmic complications associated with COVID-19: a large US national database analysis
Source: Eye (Lond). 2025 Oct 4;39(17):3148–54. doi: 10.1038/s41433-025-04050-3 (PMC12623750; doi:10.1038/s41433-025-04050-3)
Supplement: Supplementary file 4 — Supplementary Table 4 [file 41433_2025_4050_MOESM4_ESM.docx]

**Supplementary Table 4.** Propensity score matching between COVID-19 and influenza groups

| Characteristic | Mean ± SD | Patients | % of Cohort | P-Value | Std diff. |
| --- | --- | --- | --- | --- | --- |
| Age at Index | 39.3 +/- 25.5 | 77,809 | 100% | 0.001 | 0.016 |
|  | 39.7 +/- 25.8 | 77,809 | 100% |  |  |
| White |  | 51,338 | 66.00% | <0.001 | 0.019 |
|  |  | 50,652 | 65.10% |  |  |
| Unknown Race |  | 7,293 | 9.40% | <0.001 | 0.034 |
|  |  | 8,087 | 10.40% |  |  |
| Female |  | 45,752 | 58.80% | 0.68 | 0.002 |
|  |  | 45,832 | 58.90% |  |  |
| Unknown Ethnicity |  | 10,152 | 13.00% | <0.001 | 0.035 |
|  |  | 11,084 | 14.20% |  |  |
| Not Hispanic or Latino |  | 56,697 | 72.90% | 0.001 | 0.017 |
|  |  | 56,106 | 72.10% |  |  |
| Hispanic or Latino |  | 10,960 | 14.10% | 0.012 | 0.013 |
|  |  | 10,619 | 13.60% |  |  |
| Black or African American |  | 12,620 | 16.20% | 0.285 | 0.005 |
|  |  | 12,465 | 16.00% |  |  |
| Other Race |  | 3,661 | 4.70% | 0.971 | <0.001 |
|  |  | 3,658 | 4.70% |  |  |
| Asian |  | 2,397 | 3.10% | 0.414 | 0.004 |
|  |  | 2,453 | 3.20% |  |  |
| Disorders of lipoprotein metabolism and other lipidaemias |  | 31,427 | 40.40% | 0.061 | 0.009 |
|  |  | 31,065 | 39.90% |  |  |
| Diabetes mellitus |  | 17,841 | 22.90% | 0.5 | 0.003 |
|  |  | 17,953 | 23.10% |  |  |
| Overweight and obesity |  | 22,974 | 29.50% | 0.003 | 0.015 |
|  |  | 22,433 | 28.80% |  |  |
| Emphysema |  | 2,324 | 3.00% | 0.001 | 0.017 |
|  |  | 2,551 | 3.30% |  |  |
| Other chronic obstructive pulmonary disease |  | 7,736 | 9.90% | 0.016 | 0.012 |
|  |  | 8,024 | 10.30% |  |  |
| Chronic kidney disease (CKD) |  | 7,579 | 9.70% | 0.022 | 0.012 |
|  |  | 7,849 | 10.10% |  |  |
| Asthma |  | 22,645 | 29.10% | 0.412 | 0.004 |
|  |  | 22,498 | 28.90% |  |  |
| Severe acute respiratory syndrome coronavirus 2 (SARS-CoV-2) (coronavirus disease [COVID-19]) vaccine, mRNA-LNP, spike protein, preservative free, 30 mcg/0.3 mL dosage, diluent reconstituted, for intramuscular use |  | 462 | 0.60% | <0.001 | 0.093 |
|  |  | 1,210 | 1.60% |  |  |
| Severe acute respiratory syndrome coronavirus 2 (SARS-CoV-2) (coronavirus disease [COVID-19]) vaccine, mRNA-LNP, spike protein, preservative free, 100 mcg/0.5 mL dosage, for intramuscular use |  | 199 | 0.30% | <0.001 | 0.02 |
|  |  | 286 | 0.40% |  |  |
| Immunization administration by intramuscular injection of severe acute respiratory syndrome coronavirus 2 (SARS-CoV-2) (coronavirus disease [COVID-19]) vaccine, mRNA-LNP, spike protein, preservative free, 100 mcg/0.5 mL dosage; first dose |  | 217 | 0.30% | 0.962 | <0.001 |
|  |  | 216 | 0.30% |  |  |
| SARS-CoV-2 (COVID-19) vaccine, mRNA spike protein |  | 1,151 | 1.50% | <0.001 | 0.073 |
|  |  | 1,941 | 2.50% |  |  |
